# Ataxia in Neurometabolic Disorders

**DOI:** 10.3390/metabo13010047

**Published:** 2022-12-28

**Authors:** Konrad Kaminiów, Izabella Ryguła, Justyna Paprocka

**Affiliations:** 1 Students’ Scientific Society, Department of Pediatric Neurology, Faculty of Medical Sciences in Katowice, Medical University of Silesia, 40-752 Katowice, Poland; 2 Department of Pediatric Neurology, Faculty of Medical Sciences in Katowice, Medical University of Silesia, 40-752 Katowice, Poland

**Keywords:** ataxia, neurometabolic disorders, neurometabolism, movement disorders

## Abstract

Ataxia is a movement disorder that manifests during the execution of purposeful movements. It results from damage to the structures of the cerebellum and its connections or the posterior cords of the spinal cord. It should be noted that, in addition to occurring as part of many diseases, pediatric ataxia is a common symptom in neurometabolic diseases. To date, there are more than 150 inherited metabolic disorders that can manifest as ataxia in children. Neuroimaging studies (magnetic resonance imaging of the head and spinal cord) are essential in the diagnosis of ataxia, and genetic studies are performed when metabolic diseases are suspected. It is important to remember that most of these disorders are progressive if left untreated. Therefore, it is crucial to include neurometabolic disorders in the differential diagnosis of ataxia, so that an early diagnosis can be made. Initiating prompt treatment influences positive neurodevelopmental outcomes.

## 1. Introduction

A kind of movement disorder, ataxia in children is a common clinical symptom that has various origins; it presents through difficulties in the smooth and accurate execution of movements, balance disorders, and a lack of muscle control during voluntary activity [1,2,3,4,5]. Muscle strength is normal, but the coordination of the patient’s actions is disrupted, making it impossible to accurately perform activities that require the smooth interaction of several muscle groups [1,2,3,4,5]. The cause of cerebellar ataxia is most often a dysfunction of the circuit connecting the basal ganglia, cerebellum, and cerebral cortex [1,6,7]. Sensory ataxia [3,4], on the other hand, refers to a dysfunction of proprioceptive sensation correlated with peripheral nerves or with the posterior columns of the spinal cord [1,6,7]. Ataxia can manifest as gait ataxia, trunk ataxia, tremor, or nystagmus, depending on the parts of the nervous system involved [2,8,9]. It is worth noting that all types of ataxia can occur singly or together in a single patient [8].

Both acquired and inherited metabolic disorders can cause ataxia, which is one of the most common neurological manifestations of these disorders [8,10,11,12]. Neurometabolic disorders are a (clinically and genetically) heterogeneous group of rare diseases; although individually rare, they account for a large number of children who present with a spectrum of neurological symptoms in clinical practice [1,8,10,12]. Their collective prevalence is about 1 in 1000 live births [8]. Neurometabolic disorders are usually caused by complete or partial enzyme deficiencies or transporter defects, leading to clinical symptoms in the form of the accumulation of toxic products or a lack of an essential end product. These diseases have been diagnosed with increasing frequency in recent years, as a result of an intensively developing system of population-based screening (covering all newborns) and selective screening (performed on children with symptoms that may suggest the disorder) in many countries. Advances in genetics have revolutionized the way we understand, diagnose, and manage these inherited neurometabolic disorders. Thanks to these advances, a worldwide Inborn Errors of Metabolism Knowledgebase has also been established, which is a reliable source of knowledge and collects information on neurometabolic disorders presenting with ataxia (http://www.iembase.org/ (accessed on 11 November 2022)).

The central nervous system (CNS) is extremely sensitive to internal and external metabolic perturbations [13]. Regions of the brain have different metabolic and energetic requirements depending on the neuronal populations and subtypes of cells that comprise them. In particular, the cerebellum, which is made up of large Purkinje cells (the main output neurons in the cerebellum), is characterized by a high density of neurons and glial cells; an important hierarchical energy-dependent node, it has high metabolic demands and is highly susceptible to metabolic perturbations. Consequently, many neurometabolic perturbations affect the structure and, as a result, also the function of the cerebellum [13,14]. It has been shown that the aforementioned Purkinje cells have higher energy requirements than molecular and granular cells [13,15], and, as a result, they are more susceptible to energy crises. When a patient suffers from a neurometabolic disease, dysfunction, global hypoplasia, or even atrophy of the cerebellum occurs, manifesting as ataxia, eye movement disorders, speech disorders, and cognitive impairment [1,8,11,13]. Some hereditary metabolic disorders may manifest as episodic or intermittent ataxia during concurrent illness, stressful situations, prolonged fasting, or high protein intake [8,10,12,13,16,17,18]. This is inextricably linked to increased energy demand and decreased energy production due to defects in energy metabolic pathways, fuel production, or transport, or the increased production of toxic metabolites (i.e., amino acids or secondary organic acids) [8].

## 2. Materials and Methods

### 2.1. Search Strategy

The search was conducted using the Pubmed, Medline, and Google Scholar databases to identify the literature related to ataxia and neurometabolic disorders. Two authors screened the above-mentioned databases. Each database was searched individually, and search terms were applied line by line and were replicated in every source. The following terms were used for the search: “ataxia” and “movement disorders”, in combination with terms such as “neurometabolic”, “neurometabolic disorders”, “metabolic disorders”, “inherited metabolic disorders”, or “inherited”. The entire process of searching the relevant papers (the period for establishing a database of relevant articles), which was conducted by two reviewers, lasted from August 2022 to October 2022, with subsequent updates based on the latest scientific reports.

### 2.2. Study Selection and Appraisal

Manuscripts were reviewed for titles, abstracts, and entire texts based on the following criteria. The inclusion criteria were as follows: (1) original papers; (2) reviews; (3) due to the rarity of the disease, we also included case reports in our review. The exclusion criteria were as follows: (1) methodological studies, editorials, commentaries, letters, and hypotheses; (2) papers with no available abstract; (3) manuscripts in a language other than English. Titles, abstracts, and full-text articles were screened regarding the inclusion criteria by two reviewers. Next, a manual search and reference and citation tracking were undertaken by two reviewers (K.K. and I.R.) who established the final selection of papers. Any disagreement was resolved by discussion.

### 2.3. Development of the Review

The analysis was conducted according to the following steps. The first step was related to the analysis of the selected papers based on titles and abstracts; the second step was concerned with the analysis of full-text papers; and the last step included the analysis of the collected data.

## 3. How to Recognize Ataxia

### 3.1. Clinical Investigation

Clinical symptoms in patients with cerebellar ataxia are associated with impaired localization [1,8]. Dysfunction of the cerebellar vermis manifests as trunk imbalance, nystagmus, and head waddling, while impairment of the cerebellar hemispheres results in a gait that veers toward the affected side, with asymmetry of the ipsilateral extremities and a high-stepping gait [1,8,11]. The involvement of afferent/sensory ataxia manifests with a walking gait and sensory damage of the extremities. In addition, ataxia is also characterized by intention tremors and speech-forming difficulties [1].

As in any other disease, obtaining a medical history and conducting a physical examination are important and invaluable diagnostic steps. They make it possible to recognize motor abnormalities and distinguish cerebellar involvement from other affected areas of the nervous system [1,11].

The first step in the clinical evaluation of a patient with cerebellar ataxia is the diagnosis of gait imbalance, which is usually the first symptom in patients with ataxia [11,19]. Patients often struggle to climb and descend stairs; they usually have to use a handrail [1,11,13]. Other symptoms the patient may report include difficulty running, as well as leaning to one side [1]. Over time, falls are often added to this clinical picture [11,13]. In the early stages, patients may experience double vision when turning their heads quickly [1,5,11,13]. Blurred vision is also a common symptom, resulting from transient and mild double vision [1,5,11,13]. Slurred speech may occur, making it difficult to understand some words [1,11]. Patients also sometimes report loss of hand dexterity, resulting in illegible handwriting and difficulty in performing manual activities [11].

The following factors may prompt physicians to include hereditary metabolic disorders in the differential diagnosis: obtaining information in the medical history from the patient or relatives regarding the presence of recurrent episodes of lethargy or even coma during comorbidities; a history of protein aversion or lack of growth; hearing loss or significant deterioration of hearing; progressive loss of skills; hair growth abnormalities, global developmental delay (GDD), and behavioral problems [8,10]. In addition, a family history involving other family members (usually first-degree relatives) of GDD, psychiatric disorders, and cognitive impairment, as well as recurrent miscarriages in women, sudden infant death syndrome (SIDS), or congenital malformations in close family members may also be important clues to include hereditary metabolic disorders in the diagnostic process [8,10]. Although the diagnosis of ataxia is a significant first step, the symptoms associated with ataxia can often suggest a possible diagnosis [1,11]. Therefore, evaluating the patient for signs of peripheral neuropathy, autonomic symptoms, and seizures is also an important part of the clinical management of the patient [1,11]. In addition, sometimes it is also worthwhile to broaden the patient’s history by assessing their exposure to toxins and drugs, which can also prove helpful in identifying the cause [1,2,5,20,21].

In Table 1, we provide a brief summary of the clinical features associated with the most common neurometabolic disorders presenting with ataxia. Regarding the necessity of deepening our knowledge about particular neurometabolic disorders, in the references column, we provide links to studies where more information can be found.

### 3.2. Neurological Examination

The neurological examination of patients with ataxia can be divided into several parts: eyes, speech, hands, legs and gait, and typical symptoms and specific maneuvers can be very helpful in revealing pediatric ataxia [1,10,11]. The patient can be evaluated in different positions: sitting, in which the affected child manifests loss of trunk control, and walking, in which the patient demonstrates a tandem gait or deviation toward the affected side [1].

The diagnosis of ataxia is especially difficult in early childhood. The most common cerebellar symptom among children is gait instability [1]. The child stands with feet wide apart and quickly loses balance. When attempting to walk, the child sways and stops, and may also walk backwards. Some patients may have a lack of coordination of their eye movements. After the age of three, the semiology of ataxia is similar to that seen in adulthood [1].

Various abnormalities of eye movements can be associated with different types of ataxia [11]. However, it is important to note that genotypically different ataxias may have a similar clinical picture, and patients with the same genotype may have different oculomotor abnormalities. In addition, the clinical picture changes with the severity of the disease. The main anomalies include [11,83,84,85]:

- hyper- or hypometric saccades (observed in many types of ataxia);
- vertical or horizontal nystagmus (observed in many types of ataxia);
- saccadic intrusion in fixed gaze (i.e., square-wave jerks);
- breakdown of smooth pursuit;
- slow saccades;
- ophthalmoplegia/ophthalmoparesis, (observed in sensory ataxic neuropathy, dysarthria and ophthalmoparesis (SANDO));
- ptosis (observed in SANDO and ataxia associated with mitochondrial genome mutations).

Symptoms of cerebellar involvement include slurred speech, poor expression, and scarring [1,8,11,13]. Scanning speech can be common in patients with ataxia. It is characterized by a disruption of normal speech patterns and words are broken into separate syllables [1,8,11,13]. Speech speed may become slow and speech volume may be variable [1,11,13].

Maneuvers commonly used to test hand ataxia and coordination include:

- The finger–nose test (the patient repeatedly uses the index finger to touch the tip of their nose with their eyes open and then closed);
- The finger–nose test (the patient points with their index finger from the nose to the physician’s finger);
- The finger-chase test (the patient’s index finger follows the physician’s moving index finger as closely as possible);
- Rapid alternating movements (the patient performs cycles of repeated alternating pronation and supination of the hand on the thigh).

Patients with ataxia may show excessive acceleration in the finger-chase test and variable rhythm and speed in alternating movements, as well as intention tremors in the finger–nose–finger test [1,11]. This is because the tremor becomes more prominent when the fingers are closer to the target [1,11]. Another neurological test used to evaluate patients with ataxia is the heel–knee test. In this test, patients are asked to straighten one leg and use the heel of the other leg to smoothly and precisely slide the shin off the knee. Patients with ataxia will experience difficulty keeping the heel on the shin. Dysmetria observed through errors in determining the correct distance (too long or too short) can also be of diagnostic value.

Then, the patient is asked to stand in a neutral position so that the physician can observe any swaying of the trunk. Later, the patient stands with his or her feet together in tandem, on both feet, or hops on both feet. These maneuvers can highlight subtle imbalances associated with cerebellar dysfunction [1,11]. In clinical practice, the patient is often asked to close his or her eyes while performing these maneuvers. If there is a significant deterioration of balance in this situation, it indicates the involvement of sensory neuropathy [1,11,13]. During gait testing, attention should be paid to variability in the stride length and direction. It is important to remember that the features of ataxic gait can change at different stages of the disease. In mild ataxia, the gait may be narrow, but twisting in one direction and abnormal steps are often observed [1,11,13]. In moderate ataxia, the gait becomes wide to compensate for imbalance [1,11,13]; meanwhile, if the patient has more advanced ataxia, in addition to a wide-base gait, step length may be shortened to allow for further compensation [1,11,13]. For patients who have difficulty walking up stairs and on level, horizontal ground or running, the observation of their performance in such tasks will usually provide additional information for diagnosis.

Among the maneuvers used to reveal ataxia in children are the Romberg test, which is characterized by a tendency to fall down with eyes closed in the holding position, as well as the test of holding a full glass of water in the hand without spilling it [1,86].

In the Romberg test, the patient stands with feet together and arms straight, extended in front of him (elbow joints should be straight and the forearms should remain in supination), with his eyes open and then also closed. The patient should stand in this position for 30 s. It is worth noting that, when performing the Romberg test, a patient who has his eyes closed should be belayed, in order to avoid falling and suffering injury. The ability to maintain balance and the possible direction of falling are assessed: the patient falls toward the injured cerebellar hemisphere or backward in the situation of cerebellar vermis injury [1,86].

Once the presence of ataxia has been established through neurological examination, other accompanying symptoms may be key to indicating a specific diagnosis. Special attention should be paid to signs of parkinsonism, myoclonus, dystonia, tremor, hyperreflexia, sensory neuropathy and extensor reflexes [10,11]. Sensory–tactile, pain, thermal, and prosodic assessments should be recorded, and any abnormalities should be carefully checked [1].

## 4. Diagnosis of the Neurometabolic Causes of Ataxia and Their Specific Treatment

Inherited neurometabolic disorders should be included in the differential diagnosis of all children with ataxia, even if there are only a few signs and symptoms that might indicate that they could be the cause [10,87,88]. Clinicians should be particularly concerned when the causes of ataxia remain unexplained after the most common etiologies, such as drug side effects, infections, and focal brain lesions, have been ruled out [16,17,89]. Then, neurometabolic diseases should always be suspected [16,17,89]. Table 2 shows red flags suggesting that ataxia may be caused by neurometabolic diseases [10,16,17,18,89].

Due to the fact that modern science is moving away from providing information in the comprehensive form of a coherent text, neurometabolic disorders that can manifest as ataxia and the characteristics that are necessary for diagnosing clinicians are presented in the form of a table. In our opinion, this is an easier to analyze, more readable source of all the information needed by the clinician to begin to suspect a neurometabolic disorder, make a diagnosis, and initiate appropriate treatment (if available). In Table 3, we present the most common neurometabolic disorders presenting with ataxia with their characteristic biochemical abnormalities (which can be observed in the disease after the examination of blood serum, urine, or cerebrospinal fluid), abnormalities in neuroimaging studies, and disease-specific treatment (if available for the disease). Disorders that have specific treatments are highlighted by bolding their names.

Among this group of neurometabolic disorders are those that have their own characteristic clinical or neurological features. In such a situation, appropriate disease-targeted metabolic tests can be performed to provide preliminary support for the suspected diagnosis. The source of material for testing may be serum, cerebrospinal fluid (CSF), or urine. One test that is used to confirm the diagnosis of neurometabolic disorders is targeted direct Sanger sequencing [8,11]. In some cases, the patient has no specific features when they undergo clinical examination, biochemical tests, or neuroimaging. In this situation, non-targeted genetic testing, including a targeted next-generation sequencing panel, whole exome, or mitochondrial genome sequencing is used to establish the diagnosis [8,11].

## 5. Conclusions

Ataxia is an important clinical manifestation in pediatric neurology, which can pose great diagnostic difficulties due to its diverse etiology. Ataxia can occur in various types of neurometabolic disorders, including, among others, congenital disorders of the amino acid metabolism, peroxisomal disorders, congenital glycosylation disorders, organic acidosis; mitochondrial diseases, or lysosomal storage disorders.

The diagnosis of ataxia, especially in young children, can pose a considerable challenge to clinicians. Ataxia can be overlooked and erroneously associated with delayed coordination and the delayed development of the nervous system. Physical examinations with a special role for neurological examinations are extremely helpful in detecting its clinical manifestations. Once the symptoms of cerebellar ataxia are established, it is important to look for other neurological symptoms (e.g., tremor, dystonia, parkinsonism, motor neuron symptoms) as clues as to the cause of the ataxia. Clues regarding the presence of neurometabolic disorders include the presence of GDD or regression, seizures, encephalopathy, or tone abnormalities. Early diagnosis is important for both diagnostic and therapeutic purposes, as many neurometabolic disorders can be treated. For this reason, it is crucial for physicians to be familiar with the topic of neurometabolic disorders, their variety of clinical manifestations, and their underlying causes. The more we know, the easier it is to make a diagnosis and implement appropriate treatment that will limit further damage, and can improve patients’ neurodevelopmental outcomes and quality of life, and prevent premature death.

Given the rarity of neurometabolic diseases, we face the problem that only a limited number of studies have been conducted on a representative group of patients. As a result, the available evidence and management guidelines are very limited. The best resource for clinicians is prospective randomized controlled trials (RCTs), but these are difficult to conduct in diseases as rare as neurometabolic disorders. Therefore, it is important to develop a platform for knowledge sharing between researchers and clinicians. Such a platform would serve to share their insights into the causes, symptoms, diagnostic processes, and treatment options for neurometabolic diseases. Another beneficial possibility would be the formation of specialized consortia, where standardized research into the causes of and treatments for neurometabolic disorders would be carried out.

## Figures and Tables

**Table 1:** Clinical features of neurometabolic disorders presenting with ataxia.

Name	Gene	Main Age of Onset	Clinical Features	References
Vitamin and Cofactor Responsive Disorders				
Biotinidase deficiency	*BTD*	Infancy–adolescence	Ataxia, hypotonia, seizures, GDD, skin rash, conjunctivitis, alopecia, vision problems, hearing loss	[22,23]
Holocarboxylase synthetase deficiency	*HLCS*	Infancy–adolescence	Ataxia, hypotonia, seizures, GDD, lethargy, vomiting, failure to thrive, skin rash	[24]
Methylenetetrahydrofolate reductase deficiency	*MTHFR*	Adolescence–adulthood	Ataxia, hypotonia, seizures, GDD, spasticity, encephalopathy, psychiatric symptoms, cognitive dysfunction, failure to thrive, microcephaly, myelopathy, apnoea	[25,26]
Ataxia with vitamin E deficiency	*TTPA*	Childhood–adulthood	Progressive ataxia, dysdiadochokinesia, dysarthria, macular atrophy, retinitis pigmentosa, nystagmus	[27]
Cobalamin C deficiency	*MMACHA*	Childhood–adulthood	Ataxia, hypotonia, seizures, GDD, tremor, nystagmus, failure to thrive, nystagmus, pigmentary retinopathy	[28]
Riboflavin transporter deficiency neuronopathy	*SLC52A2 SLC52A3*	Infancy–adulthood	Movement disorders (i.e., ataxia, tongue fasciculations), nystagmus, failure to thrive, respiratory insufficiency, optic atrophy, sensorineural deafness,	[29]
Disorders of amino acid metabolism and transport				
Maple syrup urine disease	*BCKDHA* *BCKDHB* *DBT*	Infancy–childhood	Ataxia, seizures, GDD, failure to thrive, maple syrup smell	[30]
Nonketotic hyperglycinemia	*GLDC* *AMT*	From the neonatal period	GDD, ataxia, seizures, hypotonia, spasticity	[31]
Hartnup disease	*SLC6A19*	Childhood	Movement disorders (i.e., ataxia, dystonia, tremor), GDD, psychiatric abnormalities, nystagmus, skin rashes	[32]
Glutaminase deficiency	*GLS*	Childhood	Movement disorders (ataxia, dysarthria), hypertonia, GDD	[33]
Sulfite oxidase deficiency	*SUOX*	Neonatal–childhood	Movement disorders (ataxia, choreoathetosis, dystonia), seizures, GDD, ectopia lentis, microcephaly	[34]
Hyperornithinemia-hyperammonemia-homocitrullinuria syndrome (HHH syndrome)	*SLC25A15*	Neonatal–adulthood	Ataxia, spasticity, GDD, cognitive dysfunction, encephalopathy, chronic liver dysfunction	[35]
Isovaleric acidemia	* IVD *	Childhood	Ataxia, seizures, GDD, intellectual disability, poor feeding, emesis, lethargy, dehydration, impaired consciousness	[36]
Methylmalonic aciduria due to methylmalonyl-CoA mutase deficiency	* MMUT *	Infancy–childhood	GDD, ataxia, lethargy, hypotonia	[37]
Glutaric aciduria type 1	* GCDH *	Infancy–childhood	Ataxia, tremor, epilepsy, GDD, chronic headaches, macrocephaly	[38]
Disorders of carbohydrate metabolism				
Galactosemia	*GALT*	Adolescence–adulthood	Ataxia, speech delay, GDD, bleeding, liver failure, cataracts	[39]
Glucose transporter 1 deficiency	*SLC2A1*	Infancy–adulthood	Movement disorders (ataxia, tremor, dysarthria, chorea, dystonia), seizures, GDD, nystagmus, speech delay, acquired microcephaly	[40]
Organelle related disorders: lysosomal storage disorders				
Neuronal ceroid lipofuscinosis	*CLN1* *CLN2* *CLN5* *CLN6* *DNAJC5 MFSD8*	Childhood	Ataxia, seizures, spasticity, GDD, dementia, blindness, early death	[41]
Fabry disease	*GLA*	Childhood	Ataxia, angiokeratoma, acroparesthesia, GDD, sweating abnormalities, corneal or lenticular opacity, renal and cerebrovascular involvement, cardiac disease	[42]
Pompe disease	*GAA*	Infancy	Ataxia, hypotonia, GDD, hepatomegaly, respiratory insufficiency, cardiomegaly	[43]
Krabbe disease	*GALC*	Childhood–adulthood	Ataxia, seizures, hypotonia, spasticity, nystagmus, tremor, GDD, behavioural difficulties, peripheral neuropathy, vision loss	[44,45]
Tay–Sachs disease	*HEXA*	Childhood–adulthood	Movement disorders (ataxia, tremor, dystonia), seizures, spasticity, GDD, spasticity, vision loss, increased startle response	[46,47]
Niemann-pick type C disease	*NPC1* *NPC2*	Infancy–adulthood	Movement disorders (ataxia, tremor, dystonia, dysarthria), seziures, hypotonia, GDD, psychiatric abnormalities, hepatosplenomegaly, neonatal jaundice, dysphagia	[48,49]
Metachromatic leukodystrophy	*ARSA*	Childhood–adulthood	Movement disorders (ataxiadysarthria), seizures, hypotonia, cognitive dysfunction, GDD, psychiatric abnormalities, peripheral neuropathy spasticity, gallbladder involvement	[50,51]
Salla disease	*SLC17A5*	Infancy–childhood	Movement disorders (ataxia, athetosis), seizures, hypotonia, GDD, cognitive dysfunction, hypotonia, spasticity, facial coarsening	[52]
Sialidosis type I	*NEU1*	Childhood–adulthood	Ataxia, seizures, GDD, myoclonus, corneal opacities, vision loss,	[53]
Fatty acid hydroxylase-associated neurodegeneration	*FA2H*	Childhood–adolescence	Movement disorders (ataxia, dysarthria, dystonia), seizures, GDD, optic atrophy or oculomotor abnormalities, cognitive dysfunction	[54]
Sandhoff disease	*HEXB*	Childhood–adulthood	Ataxia, seizures, spasticity, GDD, cognitive dysfunction, exaggerated startle response, vision loss, splenomegaly	[55,56]
Disorders of lipid and bile acid metabolism				
Abetalipoproteinemia	*MTTP*	Childhood–adolescence	Ataxia, dysarthria, failure to thrive, muscle weakness, vision loss	[57]
Cerebrotendinous xanthomatosis	*CYP27A1*	Adolescence–adulthood	Movement disorders (ataxia, parkinsonism, dystonia), seizures, GDD, psychiatric disturbances, cataracts, peripheral neuropathy, dementia	[58]
Mevalonate kinase deficiency	*MVK*	Childhood	Ataxia, Failure to thrive, nystagmus, GDD, vision problems, lymphadenopathy, hepatosplenomegaly	[59]
Disorders of metal transport and metabolism				
Aceruloplasminemia	*CP*	Adolescence–adulthood	Movement disorders (ataxia, involuntary movement, dystonia, dysarthria, chorea, parkinsonism), GDD, cognitive dysfunction, diabetes mellitus, retinal degeneration, anemia	[60]
Menkes disease	*ATP7A*	Childhood	Ataxia, seizures, hypotonia, GDD, kinky hair	[61]
Organelle related disorders: peroxisomal disorders				
X-linked adrenoleukodystrophy	*ABCD1*	Childhood–adulthood	Ataxia, seizures, GDD, vision loss, behaviour problems, hearing loss	[62,63,64]
Zellweger spectrum disorders	*PEX2* *PEX10* *PEX12* *PEX16*	Childhood–adolescence	Ataxia, seizures, nystagmus, hypotonia, GDD, cognitive dysfunction, hearing loss, liver dysfunctions, retinal dystrophy, bone stippling,	[65,66]
Neurotransmitter disorders				
Succinic semialdehyde dehydrogenase deficiency	*ALDH5A1*	Childhood	Ataxia, seizures, hypotonia, GDD, behavioural problems, strabismus	[67,68]
Disorders of mitochondrial energy metabolism				
Primary coenzyme Q10 deficiency	*COQ2* *COQ4* *COQ5* *COQ6* *COQ8A* *PDSS2* *ANO10*	Childhood	Movement disorders (ataxia, dystonia, parkinsonism), seizures, spasticity, hypotonia, GDD, encephalopathy, myopathy stroke-like episodes, nephrotic syndrome, hypertrophic cardiomyopathy, retinopathy	[69,70]
Pyruvate carboxylase deficiency	*PC*	Neonatal–childhood	Ataxia, seizures, hypotonia, GDD, failure to thrive, nystagmus	[71]
Pyruvate dehydrogenase complex deficiency	*PDHA1* *PDHB* *DLAT* *PDP1*	Childhood	Ataxia, seizures, nystagmus, hypotonia, spasticity, GDD, microcephaly, encephalopathy, peripheral neuropathy	[72,73]
Dihydrolipoamide dehydrogenase deficiency	*DLD*	Childhood	Ataxia, seizures, hypotonia, spasticity, tremor, GDD, microcephaly, vision impairment, hepatomegaly, liver dysfunction	[74]
GAMT deficiency CRTR deficiency	*GAMT* *SLC6A8*	Childhood Infancy–childhood	Movement disorders (ataxia, dystonia, chorea), seizures, hypotonia, GDD, cognitive dysfunction, behavioural disorder, speech delay, dysmorphic features (in *SLC6A8*)	[75,76] [77,78]
MELAS	*MT-TL1* *MT-ND5*	Childhood–adulthood	Ataxia, seizures, GDD, stroke-like episodes, recurrent headaches, dementia, hearing impairment, peripheral neuropathy, ragged red fibers on muscle biopsy	[79]
Kearns–Sayre syndrome	*mtDNA deletion*	Childhood–adolescence	Ataxia, GDD, cognitive dysfunction, cardiac conduction abnormality, progressive external ophthalmoplegia, pigmentary retinopathy, hearing loss	[80]
Congenital disorders of glycosylation (CDG)				
PMM2-CDG	*PMM2*	Infancy–childhood	Ataxia, seizures, hypotonia, nystagmus, GDD, peripheral neuropathy, strabismus, endocrine system dysfunction, skin and skeletal abnormalities	[81,82]
ALG6-CDG	* ALG6 *	Neonatal–adulthood	Ataxia, dysarthria, dysmetria, hypotonia, myasthenia, peripheral neuropathy	[82]
ALG1-CDG	*ALG1*	Childhood	Ataxia, seizures, hypotonia, nystagmus, GDD, intellectual disability, strabismus, peripheral neuropathy	[82]
PIGG-CDG	*PIGG*	Childhood	Ataxia, seizures, hypotonia, GDD, strabismus, peripheral neuropathy,	[82]
ST3GAL5-CDG	*ST3GAL5*	Childhood	Ataxia, seziures, hypotonia, GDD, intellectual disability	[82]

**Abbreviations:** GDD = global developmental delay; GAMT = guanidinoacetate methyltransferase; CRTR = creatine transporter.

**Table 2:** Red flags in ataxia pointing to a potential neurometabolic etiology [10,16,17,18,89].

Early age of onset (the earlier the onset of symptoms, the greater possibility that a neurometabolic disorder is the cause)
Diffuse clinical picture with several neurological and non-neurological symptoms or progressive neurodegeneration
Distinct neuroradiological findings, e.g., basal ganglia abnormalities, white matter involvement, or cerebellar atrophy
Atypical or progressive ataxia that does not respond to standard treatment
Combination of ataxia with other movement disorders (e.g., dystonia, parkinsonism, chorea, or myoclonus)
Acute or subacute onset, especially if the onset is associated with encephalopathy, or if the onset is accelerated by a comorbid illness with fever, starvation, or physical exhaustion, or after ingesting large amounts of protein
An insidious onset in a patient with multiple previous extra-systemic symptoms
Paroxysmal episodic events
Autonomic dysfunction
Relatedness among individuals with similar symptoms (family history of similar disorders)

**Table 3:** Neurometabolic disorders presenting with ataxia: biochemical abnormalities, neuroimaging abnormalities, and disease-specific treatments.

Disease	Biochemical Abnormalities	Neuroimaging Abnormalities	Specific Treatment	Ref.
Biotinidase deficiency	↓ Serum biotinidase activity ↑ 3-methylcrotonylglycine, 3-hydroxyisovaleric acid, methylcitrate, 3-hydroxypropionate in urine organic acid analysis Metabolic ketolactic acidosis Hyperammonemia	Normal to myelopathy changes, cerebral or cerebellar atrophy	Biotin	[22,23]
Holocarboxylase synthetase deficiency	↑ hydroxypentanoylcarnitine ↑ 3-methylcrotonylglycine, 3-hydroxyisovaleric acid, methylcitrate, 3-hydroxypropionate in urine organic acid analysis Metabolic ketolactic acidosis	Normal to myelopathy changes, cerebral atrophy,	Biotin	[24]
Methylenetetrahydrofolate reductase deficiency	↑ homocysteine in plasma ↓ to normal methionine in plasma	Normal to stroke and/or WM changes (increased WM signal in T2), possible brain atrophy	Betaine, folic acid, methionine, pyridoxine, carnitine, 5-methyltetrahydrofolate	[25,26]
Ataxia with vitamin E deficiency	↓ vitamin E level	Normal to cerebellar atrophy	Vitamin E supplementation (p.o.)	[27]
Cobalamin C deficiency	↑ homocysteine in plasma ↓ to normal methionine in plasma ↑ methylmalonic acid in urine	Brain atrophy, WM edema	Hydroxocobalamin, betaine, carnitine, folic acid	[28]
Riboflavin transporter deficiency neuronopathy	Abnormalities in acylcarnitine profile (↑ short to long chain species)	Normal to cerebellar atrophy	Riboflavin	[29]
Maple syrup urine disease	↑ leucine, alloisoleucine, isoleucine, valine in plasma ↑ ketones and metabolic acidosis during acute decompensation	Increased signal and cytotoxic edema myelinated structures, vasogenic edema of unmyelinated tracts	Leucine-restricted diet, BCAA diet restriction	[30]
Nonketotic hyperglycinemia	↑ plasma glycine ↑ CSF glycine abnormal CSF-to-plasma glycine ratio	Hypogenesis of the CC, T2 hyperintensities and DWI restriction of myelinated tracts	Benzoate, NMDA receptor antagonist	[31]
Hartnup disease	↑ alanine, threonine, serine, leucine, valine, isoleucine, tyrosine, histidine, phenylalanine, glutamine, tryptophan, asparagine, citrulline in urine	Normal to diffuse brain atrophy	Nicotinamide, neomycin, tryptophan ethyl ester, tryptophan rich protein intake	[32]
Hyperornithinemia-hyperammonemia-homocitrullinuria syndrome (HHH syndrome)	hyperammonemia and hyperornithinemia in plasma urinary excretion of homocitrulline	Normal to mild cerebral and cerebellar atrophy, white matter changes, subdural hematoma, cystic lesions and calcifications, and diffuse brain edema	low-protein diet supplemented with citrulline or arginine Protein restriction may be combined with sodium benzoate or sodium phenylbutyrate	[35]
Isovaleric acidemia	↑ Urine 2-methylbutyrylglycine ↑ Plasma leucine and carnitine	Normal to basal ganglia T2 hyperintensity	Glycine, carnitine suplementation	[36]
Methylmalonic aciduria due to methylmalonyl-CoA mutase deficiency	↑ methylmalonic acid (MMA) concentration in the blood and urine hyperammonemia, Severe ketoacidosis and lactic acidosis (plasma) ↑ urine organic acids	Normal to basal ganglia involvement	Low protein diet, carnitine, hydroxycobalamin suplementation	[37]
Glutaric aciduria type 1	↑ concentrations of glutaric acid, 3-hydroxyglutaric acid, glutarylcarnitine (C5DC), glutaconic acid	Basal ganglia involvement	Carnitine, lysine-restricted/argininerich diet	[38]
Galactosemia	↑ erythrocyte galactose-1-phosphate ↓ erythrocyte GALT activity	Normal to cerebral and cerebellar atrophy	Galactose and lactose restriction, vitamin D, calcium	[39]
Glucose transporter 1 deficiency	↓ glucose in CSF with normal glucose in plasma ↓ erythrocyte 3-O-methyl-D-glucose uptake	Normal	Ketogenic diet, triheptanoin	[40]
Neuronal ceroid lipofuscinosis type 2 *CLN2* disease	↓ tripeptidyl peptidase 1 activity	Cerebral and cerebellar atrophy	Cerliponase alfa intracerebroventricular	[41]
Fabry disease	↓ alpha-galactosidase A activity ↑ globotriaosylsphingosine in plasma and urine	Cerebral atrophy, increased signal intensity in WM in T2, stroke-like lesions	Agalsidase beta	[42]
Krabbe disease	↓ galactocerebrosidase activity	Cerebral atrophy, demyelination in brain stem and cerebellum, leukodystrophy, chiasmatic enlargement	Hematopoietic stem cell transplant	[44,45]
Tay-Sachs disease	Absence or extremely low level of hexosaminidase activity (0–5%) ↓ Hexosaminidase levels in the serum	Normal to cerebellar atrophy	-	[46,47]
Niemann-pick type C disease	↑ oxysterols in plasma Positive filipin staining in cultured fibroblasts	Cerebral and cerebellar atrophy, increased WM intensity in T2	Miglustat	[48,49]
Metachromatic leukodystrophy	↓ arylsulfatatase A activity ↑ sulfatides in urine	Cerebral atrophy, demyelination in brain stem and cerebellum, leukodystrophy, chiasmatic enlargement	Hematopoietic stem cell transplant	[50,51]
Salla disease	↑ Sialic acid, free in urine	Delayed myelination	-	[52]
Sialidosis type I	Sialyloligosaccharides in urine, deficiency of the lysosomal sialidase activity in cultured skin fibroblasts obtained from a skin biopsy	Normal to cerebellar atrophy	-	[53]
Fatty acid hydroxylase-associated neurodegeneration	-	Iron deposition in basal ganglia and WM changes	-	[54]
Abetalipoproteinemia	↓LDL-cholesterol, ↓ triglyceride ↓ apolipoprotein B	Normal to delayed myelination	Low-fat diet, supplementation of essential fatty acid, vitamin A, D, E, K supplementation	[57]
Cerebrotendinous xanthomatosis	↑ cholestanol in plasma ↓ to normal cholesterol in plasma	Diffuse brain atrophy	CDCA	[58]
Aceruloplasminemia	↓ ceruloplasmin in serum ↓ serum copper or iron ↑ ferritin in serum ↑ hepatic iron	Decreased signal intensity in BG in T2, Iron deposition in basal ganglia	Desferrioxamine, deferasirox, deferiprone, combined intravenous desferrioxamine and FFP	[60]
Menkes disease	↓ serum copper concentration ↓ serum ceruloplasmin concentration	Arterial tortuosity and/or subdural collection	Copper histidine	[61]
X-linked adrenoleukodystrophy	↑ VLCFA in plasma	Symmetric enhanced T2 signal in the parieto-occipital region with contrast enhancement at the advancing margin	Hematopoietic stem cell transplant	[62,63,64]
Succinic semialdehyde dehydrogenase deficiency	↑ urine organic acids (↑ 4-hydroxybutyric acid concentration) ↑ plasma 4-hydroxybutyric acid concentration	Globus pallidus, dentate, and subthalamic nucleus T2 hyperintensit	-	[67,68]
Primary coenzyme Q10 deficiency	↓ coenzyme Q10 in skeletal muscle ↓ complex I + III and II + III activity in muscle	Normal to cerebellar atrophy, and increased T2 signal intensity cerebellum	Coenzyme Q10	[69,70]
Pyruvate carboxylase deficiency	Normal lactate to pyruvate ratio ↑ alanine, citrulline, lysine in plasma and urine ↓ aspartic acid, glutamine in plasma and urine ↑ lactate	Hypomyelination, periventricular cysts in cortex, BG, brain stem, and cerebellum	Acute management: IV glucose Chronic management: citrate, aspartate, biotin, liver transplantation	[71]
Pyruvate dehydrogenase complex deficiency	↑ lactate in CSF and blood ↑ pyruvate and alanine in CSF and blood ↑ ***α*** -ketoglutarate in urine ↑ leucine, valine, isoleucine, alloisoleucine in plasma	Brain atrophy and/or basal ganglia T2 hyperintensity	Ketogenic diet, thiamine	[72,73]
Dihydrolipoamide dehydrogenase deficiency	↑ Blood and CSF lactate ↑ Blood and CSF pyruvate and alanine ↑ alpha ketoglutarate in urine organic acid analysis ↑ leucine, valine, isoleucine, alloisoleucine in plasma amino acid analysis	Basal ganglia T2 hyperintensity	Thiamine, ketogenic diet	[74]
Cerebral creatine deficiency syndromes	↑ urine, plasma GAA (*GAMT* deficiency) ↑ urine creatine to creatinine ratio	Normal to increased T2 signal in BG	*GAMT* deficiency: creatinine, ornithine, arginine restricted diet *CRTR* deficiency: arginine, glycine, creatine	[75,76,77,78]
MELAS	-	Stroke-like episodes	-	[79]
PMM2-CDG	↑ Serum sialotransferins, Coagulopathy and thrombosis (factor II, V, VII, VIII, IX, X, XI, antithrombin III, protein C, protein S deficiency	Cerebellar atrophy	-	[81,82]
ALG6-CDG	↑ Serum sialotransferins, Coagulopathy and thrombosis (factor II, V, VII, VIII, IX, X, XI, antithrombin III, protein C, protein S deficiency	Normal to cerebral and/or cerebellar atrophy	-	[82]
PIGG-CDG	GPI-anchored protein flow cytometry	Cerebellar atrophy	-	[82]
ST3GAL5-CDG	No laboratory tests --> DNA	Normal	-	[82]

**Abbreviations:** Ref. = references; bold name = disease that has specific treatment; ↑ = elevated; ↓ = decreased; WM = white matter; BCAA = branched chain amino acid; BG = basal ganglia; CLN2 = Neuronal ceroid lipofuscinosis type 2; CDCA = Chenodeoxycholic acid; FFP = fresh-frozen human plasma; CRTR = Creatine Transporter; CSF = cerebrospinal fluid; GAA = guanidinoacetate; GALT = galactose-1-phosphate uridylyltransferase; GAMT = guanidinoacetate methyltransferase; VLCFA = very long chain fatty acids.
